# *FTO* Gene Polymorphisms at the Crossroads of Metabolic Pathways of Obesity and Epigenetic Influences

**DOI:** 10.17113/ftb.61.01.23.7594

**Published:** 2023-03

**Authors:** Ana-Marija Popović, Ana Huđek Turković, Kristina Žuna, Višnja Bačun-Družina, Ivica Rubelj, Martina Matovinović

**Affiliations:** 1 General Hospital Gospić, Kaniška 111, 53000 Gospić, Croatia; 2 Faculty of Food Technology and Biotechnology University of Zagreb, Pierottijeva 6, 10000 Zagreb, Croatia; 3 Ruđer Bošković Institute, Bijenička 54, 10000 Zagreb, Croatia; 4 Clinical Hospital Centre Zagreb, Kišpatićeva 12, 10000 Zagreb, Croatia

**Keywords:** obesity, *FTO* gene, single nucleotide polymorphisms, epigenetic influence

## Abstract

In this review, we summarize the current state of knowledge on the fat mass and obesity-associated (*FTO*) gene and its role in obesity. The FTO-encoded protein is involved in multiple molecular pathways contributing to obesity as well as other metabolic complexities. This review emphasizes the epigenetic influence on the *FTO* gene as a new approach in the treatment and management of obesity. Several known substances have a positive effect on reducing *FTO* expression. Depending on which variant of the single nucleotide polymorphism (SNP) is present, the profile and level of gene expression changes. Implementation of environmental change measures could lead to reduced phenotypic manifestation of *FTO* expression. Treating obesity through *FTO* gene regulation will have to include various complex signal pathways in which *FTO* takes part. Identification of *FTO* gene polymorphisms may be useful for the development of individual obesity management strategies, including the recommendation of taking certain foods and supplements.

## INTRODUCTION

The World Health Organization (WHO) defines obesity as an excessive or abnormal increase in fat mass with a body mass index (BMI)≥30 kg/m^2^ and a causal factor in development of health problems such as diabetes mellitus, cardiovascular diseases and various types of cancer (*1*). Over 1.9 billion adults were overweight of which more than 650 million people were obese in 2016 and that number is expected to increase to 1.12 billion by 2030. The problem increases if obesity occurs at an early age. The fact that in 2020 there were 39 million obese children further emphasizes the seriousness of the problem (*2*). This has become a major problem in both developed as well as developing countries.

Environmental factors such as physical inactivity, stress, low-nutrient diets and various microbial and chemical exposures contribute to the development of obesity (*3*, *4*). Obesity is not only a result of environmental factors, but also of individual genetic predispositions (*5*). Between 30 and 70% of common obesity is hereditary (*3*). Some of the gene-dependent types of obesity are monogenic, syndromic, oligogenic and polygenic obesity. The underlying mechanism behind polygenic obesity is complex, with complicated interactions between genes themselves and gene-environment interactions. Other types of gene-dependent obesity are very rare, with monogenic obesity depending only on genetic influences (*6*).

Previous genome-wide association studies revealed a relationship between the *FTO* gene and obesity. Changes particularly located in the cluster of single nucleotide polymorphisms (SNPs) in the first intron of the *FTO* gene, for example, rs9930506, were associated with the changes in BMI. The cluster of SNPs on chromosome 16 includes rs9939609 and rs9926289, which are found in an intronic region of the *FTO* gene that is highly conserved across species (*7*). This research includes epigenetic influences on *FTO* variants and reports a combined impact of environmental factors such as lifestyle and food consumption. The aim of this review is to report new mechanisms affecting *FTO* expression and to reveal new personalized paths in treating obesity that could be scaled globally. Since the treatment of obesity in most cases includes the treatment of other diseases, there is a need to find a common approach with an emphasis on epigenetics.

## FTO PROTEIN

### Molecular function and structure

**Fig. 1:**
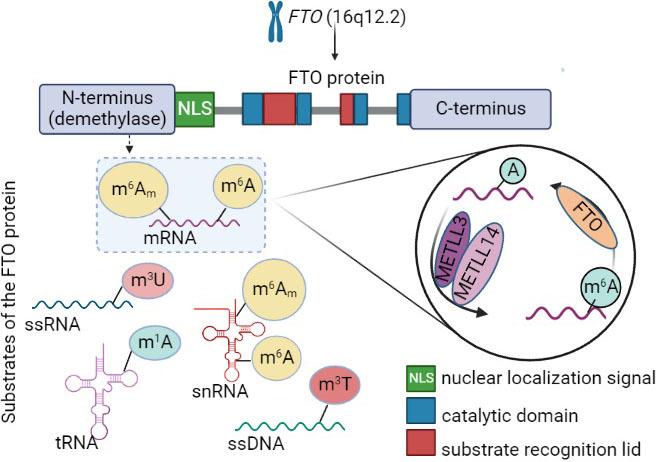
Position of *FTO* gene and molecular pathway of FTO protein. N-terminus of the FTO protein has demethylation activity on various FTO substrates in the RNA and DNA. Demethylation of the m^6^A and m^6^Am takes place *via* FTO, and re-established over METLL3 and METLL14. m^6^A_m_=*N*^6^,2’O-dimethyladenosine, m^6^A=*N*^6^-methyladenosine, mRNA=messenger RNA, snRNA=small nuclear RNA, m^3^U=3-methyluracil; ssRNA=single stranded RNA, m^1^A=*N*^1^-methyladenosine, tRNA=transfer RNA, m^3^T=3-methylthymine, ssDNA=single stranded DNA, METLL3=methyltransferase-like 3, METLL14=methyltransferase-like 14, *FTO*=fat mass and obesity-associated gene

The *FTO* gene is located on the long arm of chromosome 16, in the region 16q12.2. It is over 400 kb long and contains 9 exons and 8 introns (*8*). Sequence analysis has shown that the FTO protein has a double-stranded beta helix fold (*9*). The crystal structure of the FTO protein reveals Fe(II) and alpha-ketoglutarate dependent activity at the N-terminus of the FTO. The product of the *FTO* gene is the fat mass and obesity-associated protein, the first identified RNA demethylase. The FTO protein is composed of an N-terminal domain (NTD, amino acid residues 32–326) known to have oxygenase/demethylase activity, and a C-terminal domain (CTD, amino acid residues 327–498), whose function is primarily manifested in the stabilization of the NTD structure (*10*–*12*). It has recently been reported that the function of the C-terminal domain may involve interaction with other proteins in order to provide specific interactions for gene regulation. The FTO protein is an alpha-ketoglutarate-dependent oxygenase with a conserved jelly-roll motif. Unlike other proteins in the same family, such as alpha-ketoglutarate-dependent dioxygenase (AlkB), this protein has an extra loop covering one side of its structure that plays an important role in the selection of the FTO against double-stranded nucleic acids (*10*). Another difference between the FTO and AlkB is that FTO contains a C-terminal end and has a K216 residue (lysine) located on a long loop named the 'FTO unique loop' (*11*). The RNA-binding protein splicing factor proline- and glutamine-rich (SFPQ) enables the selection and demethylation of specific FTO substrates. The activity of the *FTO* gene and demethylation pathway of FTO substrates are described in Fig. 1. As the SFPQ is located close to the RNA-binding sites, it creates bonds with the CUGUG motif, engages FTO and promotes proximal *N*^6^-methyladenosine (m^6^A) demethylation (*13*).

Further analysis of FTO revealed two glutamine residues, Q86 (substrate sequence recognition) and Q306 (binding affinity) located on its short loop. Mutation of glutamines into lysines resulted in a stronger binding affinity of *FTO* towards ssDNA. Moreover, double mutation of glutamine residues results in an even stronger binding affinity, approx. 16-fold increase in comparison to wild-type *FTO*, while mutations in the catalytic pocket decrease the binding affinity. The catalytic pocket stabilises the methyl group through interactions between hydrogen bonds with residues R96 (N^1^ atom) and E234 (N^6^ and N^7^ atoms) (*11*). If polymorphisms are located at sites important for activity and function, they could affect the binding affinity and thereby contribute to obesity by upregulating other genes.

FTO demethylases 1-methyladenine (1-meA), 1-methylguanine (1-meG), 3-methylcytosine (3-meC), and 3-methylthymine (3-meT) on single-stranded nucleic acids (*14*). In RNA, FTO can demethylase 3-methyluracil (3-meU) and *N*^6^-methyladenosine (m^6^A) (*9*). The FTO has a 50-fold greater affinity for m^6^A than for 3-meU. The 3-meU is mainly found in ribosomal RNA (*15*), whereas m^6^A is found in mRNA.

The function of FTO manifests as demethylase activity at the most prevalent RNA modification, m^6^A, normally located around the 3' UTR region and stop codon (*16*), thereby regulating the expression of certain target genes. The well-established mechanism of epitranscriptomic modification includes writers: methyltransferase-like 3 (METTL3) and methyltransferase-like 14 (METTL14), erasers (FTO) and alpha-ketoglutarate-dependent dioxygenase AlkB homolog 5 (AlkB5) and readers YTH N6-methyladenosine RNA-binding protein 2 (YTHDF2) and YTH *N*^6^-methyladenosine RNA-binding protein 3 (YTHDF3).

Recent studies have revealed new insights into demethylation activity and substrate binding with even m^6^A being considered an *FTO* substrate; however, evidence points to a new substrate of *FTO*, *N*^6^,2’-O-dimethyladenosine (m^6^A_m_), which has 100-fold greater demethylation activity. These two substrates share structural similarities as they both have a methyl group on the sixth carbon atom of adenine ring. Transcripts that have m^6^A_m_ are protected from degradation by the mRNA-decapping enzyme 2 (DCP2) because m^6^A_m_ displays greater resistance to DCP2 and it is located in m^7^G on 5' end of the mRNA (*17*). As mentioned previously, mutations within the catalytic pocket decrease binding affinity, whereas mutations of the E234 residue responsible for the engagement with the N^6^ atom showed no significant changes in the binding activity. It is therefore a reasonable assumption that the substrate specificity is a result of intervention between the nucleobase and residues within the catalytic pocket (*11*).

The FTO is localized in the nucleus and it is thought to demethylate m^6^A during transcription and make changes before the mRNA is exported to the cytoplasm (*14*, *18*). A recent study reported that FTO was also found in the cytoplasm in a tissue-specific expression. The FTO protein was found in the cytoplasm of the cells of adipose tissue, pancreas, liver and salivary glands as analysed by Western blot analysis. Pancreatic FTO protein was found only in islets of Langerhans, which could be associated with glucose intolerance. It was also reported that FTO correlates with age, observing a decrease in the FTO protein levels in skeletal muscle when comparing neonates and 11-month-old pigs. Decreased levels of FTO were also found in the thyroid gland and adipose tissue (*19*). FTO regulates expression through transcription activity of neighbouring genes and acts directly or indirectly on various signal pathways (*e.g.* mTORC1/AMPK).

All reported results elucidate FTO function and substrate binding while suggesting substantial FTO activities. However, more detailed studies are needed in order to precisely determine the exact molecular regulation and physiological mechanism that FTO exhibits in gene regulation.

### *FTO* gene expression

The *FTO* is an ubiquitously expressed gene, as demonstrated by studies in both laboratory rats and humans (*20*, *21*). Although *FTO* is mainly expressed in the cytoplasm of the cell, it has been found to be expressed in nuclear speckles as well (*22*). Because of its N-terminal domain, the FTO protein is able to shuttle between the nucleus and cytoplasm by binding to the exportin protein XPO2 (*23*).

### *Fto* expression in rodents

Increased *Fto* expression in mice leads to obesity *via* hyperphagia. Mice with three or four copies of the *Fto* gene showed increased food intake and body mass regardless of the diet. Mice with increased *Fto* expression developed glucose intolerance when fed a high-fat diet (*24*).

Germline knockout of the *Fto* gene in mice results in growth retardation, a leaner phenotype and increased energy expenditure (*25*, *26*). However, in adult mice, loss of *Fto* expression resulted in normal growth but reduced lean mass and increased fat mass (*25*). Levels of the Fto are the highest in the central nervous system, particularly in the feeding-related nuclei in the hypothalamus (*27*). Deletion of the *Fto* in central nervous system (CNS) of mice resulted in an increase in daily energy expenditure accompanied by physical changes, consistent with the role of the Fto in hypothalamus involved in the regulation of food intake. Another finding of that study is that specific *Fto* knockout mice had lower bone density than control mice (*28*), while overexpression of the *Fto* gene causes obese or overweight mice (*27*, *29*).

**Fig. 2:**
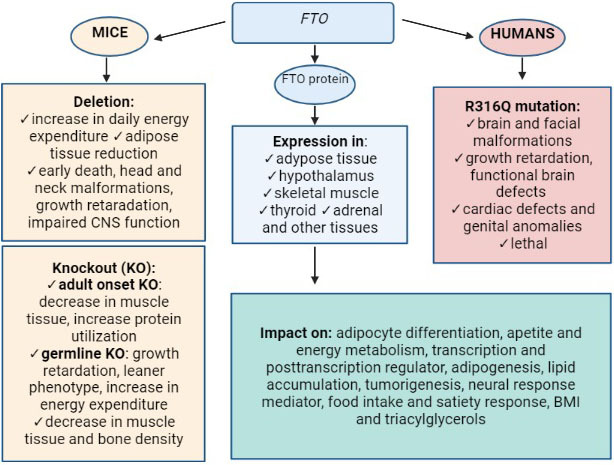
Schematic illustration of *FTO* expression in humans and mice. CNS=central nervous system, BMI=body mass index, KN=knockout

Homozygous deletion of *Fto* in mice leads to death during embryonic development with severe malformations of the head and neck, while heterozygotes exhibit malformations such as fused fingers and enlargement of the thymus (*30*). Specific deletion of the *Fto* in the CNS has a similar phenotype as well as does a whole body deletion (reduced adipose tissue, increased food consumption); however, it retains the same effect for postnatal development (*29*). Mouse models of *Fto* deficiency show its importance in neural development and retardation. An example is *Fto*^-/-^ null mice with a complete lack of Fto protein which results in mass loss by 30–40%, stunted growth and early death (*29*, *30*). The amino acid substitution mutation *Fto*^I367F^ (isoleucine to phenylalanine at position 367) leads to a decrease in catalytic protein activity with a 10% reduction in body mass in adulthood. Only mice with complete protein deficiency manifest growth retardation and death, indicating that partial Fto function is sufficient to abrogate the phenotype observed in *Fto* null mice (Fig. 2) (*30*).

Energy balance is regulated by the brain, specifically the hypothalamus, which implies that FTO plays an important role in regulating metabolism and eating habits (*31*). However, it is still unknown in which way FTO influences the changes in neuronal activity, either directly through changes in the *FTO* expression or indirectly by influencing the release of messenger molecules and/or hormones from cells (*22*). McTaggart *et al*. (*22*) found that in mice, Fto levels are relatively uniform in the regions of the hypothalamus, cerebellum and rostral brain, and are higher in the brain than in the lower skeletal muscles.

*FTO* gene expression in the brain was also determined after various periods of fasting by measuring mRNA and protein levels. In rats, fasting for 48 hours resulted in an overall increase in the mRNA and protein levels in the hypothalamus, but not uniformly in all regions (*32*). However, some studies found that >40-hour fast in mice did not significantly alter mRNA levels (*33*) or decrease in the hypothalamus (*14*). Short term fasting (16 h) in mice reduced mRNA levels in the hypothalamus (*34*).

Current studies have shown that the number of calories and amount of ingested macronutrients can influence *FTO* expression in the hypothalamus. However, FTO is thought to have many functions in the hypothalamus, so the exact relationship between the intake of specific macronutrients and the optimal changes in the *FTO* expression to maintain a healthy mass is not yet known (*35*).

The *FTO* gene is also expressed in adipose tissue. A 2015 study showed that FTO affects fat mass accumulation by regulating adipocyte differentiation *in vivo,* as demonstrated by experiments with mice overexpressing the *Fto* gene and mice in which the *Fto* gene was deleted (*36*).

### *FTO* expression in humans

In 2013, Bravard *et al*. (*37*) studied the expression of *FTO* in subcutaneous and omental adipose tissues in lean (mean BMI 24.7 kg/m^2^) to moderately obese women (mean BMI 25.8-26.5 kg/m^2^). *FTO* expression was higher in omental than in subcutaneous adipose tissue. Expression in subcutaneous tissue was associated with insulin sensitivity, and expression in omental adipose tissue with adiposity. One study found that *FTO* expression was higher in separated and isolated adipocytes than in subcutaneous adipose tissue, implying that *FTO* expression is higher in adipose tissue than in the stromal vascular cells (*33*).

Recent studies in mice (*14*, *22*, *32*–*34*) have also shown *FTO* expression in brain, especially in the hypothalamus. In humans, *FTO* is also highly expressed in the regions of the hypothalamus, particularly in its arcuate, paraventricular, dorsomedial and ventromedial nuclei (*14*), which are associated with the regulation of appetite and energy metabolism. *FTO* expression in these nuclei may vary, possibly due to different *FTO* genotypes (*38*), but the differences could also occur due to various exercise habits among individuals. A previous study in mice has demonstrated that exercise training can lead to weaker association between the *FTO* and the development of obesity (*39*). Some *in vivo* studies on human brains were performed using functional magnetic resonance imaging (fMRI) and found that *FTO* expression is higher in the prefrontal cortex after food intake (*40*). Homozygous carriers of the *FTO* rs9939609 risk allele A showed different results when examined with fMRI due to a decrease in ghrelin concentrations upon food intake (*41*). However, there are few studies of *FTO* expression in the human brain (*in vivo* or post-mortem) that could shed light on how *FTO* expression becomes altered in the brain in relation to food intake or different *FTO* genotypes.

Furthermore, *FTO* is expressed in skeletal muscle cells, but its expression is not associated with fat mass fraction or BMI, and is positively associated with glucose oxidation rate and expression of genes involved in oxidative phosphorylation (*42*). *FTO* is also expressed in the human placenta and may play a role in the regulation of foetal body mass, but is not associated with the placental SNP rs9939609 (*43*).

Physical intervention and special diet in obese individuals resulted in a reduction in anthropometric measurements along with an increase in the *FTO* expression and a positive correlation with the increase in fat-free mass (*44*). In previously reported studies, increased *FTO* expression was associated with mass gain and higher BMI. However, the *FTO* SNPs that affect metabolism could be epigenetically influenced in several ways, so further studies should include genotype as one of the most important variables in epigenetic evaluation.

A previously unknown function of the *FTO* is the newly reported role in osteoporosis, as in humans *FTO* in rs1121980 variant is associated with risk of hip fracture (*45*). FTO activity speaks in favour of the fact that there may be hidden novel mechanisms. These findings suggest an important physiological role in both the brain and other tissues related to metabolic mechanisms. As it appears, a complete loss of FTO is required for damage to the osteoblast function. FTO has been shown to be essential for muscle and thyroid function as the lack of enzymatic activity at key sites in the DNA repair pathway makes cells more susceptible to damage and apoptosis (*46*). A schematic illustration of *FTO* expression or defect in humans and mice summarizing its main roles is shown in Fig. 2.

Loss of function in humans results from the R316Q mutation, arginine to glutamine substitution (Arg316Glu), phenotypically represented with severe brain malformations, psychomotor delay, functional brain defects, postnatal psychomotor delay, facial and brain dimorphism, cardiac and genital defects (*30*). This recessive autosomal mutation with lethal syndrome is the result of a catalytically inactive protein. Fibroblasts obtained from affected families displayed reduced proliferation and hastened senescence (*26*, *47*). Loss-of-function mutation in humans is equally represented as in *Fto* (*Fto^-/-^*) null mice (*29*). In addition to retardation, R316Q mutation-affected individuals have CNS abnormalities and defects in the cardiovascular system (*30*).

### Functional role of *FTO* gene polymorphisms in metabolic pathology through interaction with other genes

The interaction of the *FTO* gene SNPs with the Iroquois homeobox 3 (*IRX3*) and Iroquois homeobox 5 (*IRX5*), genes also associated with the development of obesity and an effector of the *FTO* variants (*48*), may jointly regulate adipogenesis and cause white adipose tissue browning in mice (*49*).

**Fig. 3:**
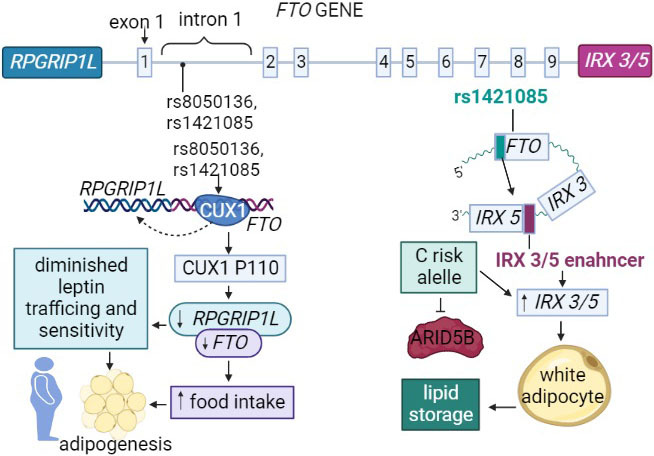
Molecular interactions between the *FTO* single nucleotide polymorphisms (SNPs) and neighbouring genes. SNP rs8050136 A allele and rs1421085 C allele have reduced affinity for CUX1 P110 isoform, resulting in the reduced activity of the *FTO* and *RPGRIP1L*, causing diminished response to leptin. SNP rs1421085 affects ARID5B repressor responsible for the activity of the *IRX3* and *IRX5* genes, involved in adipocyte differentiation. *RPGRIP1L*=retinitis pigmentosa GTPase regulator-interacting protein-1-like, *FTO*=fat mass and obesity-associated gene, *IRX3*, *IRX5* and *IRX3/5*=Iroquois homeobox 3, 5 and 3/5 respectively, CUX1=cut-like homeobox 1, ARID5B=AT-rich interaction domain 5B

The rs1421085 *FTO* polymorphism disrupts a conserved motif for AT-rich interaction domain 5B (ARIDB5) repressor binding, resulting in increased gene expression of *IRX3* and *IRX5*, which encode proteins involved in adipocyte differentiation. Increased expression of *IRX3* and *IRX5* leads to the development of white adipocytes that store energy (*50*). Also, rs1421085 allele C and rs8050136 allele A have reduced affinity for cut-like homeobox 1 (*CUX1*). The rs8050136 displayed decreased affinity for the P110 isoform of CUX1, which should increase the transcription of the *FTO* and retinitis pigmentosa GTPase regulator-interacting protein-1-like (*RPGRIP1L*) genes. The P110 isoform is expressed in hypothalamus and, when the rs8050136 variant A is present, activation of *FTO* and *RPGRIP1L* is reduced (*50*, *51*) and therefore leads to an impaired cellular response to leptin. The *RPGRIP1L* encodes a protein expressed in cilia. Cilia are organelles in eukaryotes, present in various tissues, including brain, hippocampus and hypothalamus, and belong to the leptin receptors isoform β grouping. Interactions between the *FTO* SNPs and neighbouring genes are described in Fig. 3.

The rs8050136 *FTO* polymorphism is in a haplotype with increased DNA methylation containing highly conserved noncoding elements (HCNE), which is actually a long-range enhancer (*52*). Alterations in that region can affect many tissues because the *FTO* interacts with various genes. Notably, *FTO* gene has many enhancers within which there are different variants of *FTO* gene that have an effect on tissues or transcription levels.

The rs9939609 *FTO* polymorphism is associated with obesity, with more pronounced features as the risk allele increases. Furthermore, it is likely to assume that higher levels of methylation allow for a stronger influence of polymorphism. Namely, the AA genotype has a higher risk of obesity along with higher *FTO* methylation levels than the same genotype but with lower levels. Also, higher methylation levels in the risk allele carriers are associated with shorter telomeres (*53*). Thus, it can be concluded that methylation plays an important role in the *FTO* gene expression. However, the influence of the polymorphism itself on other obesity-related genes has also been reported.

**Fig. 4:**
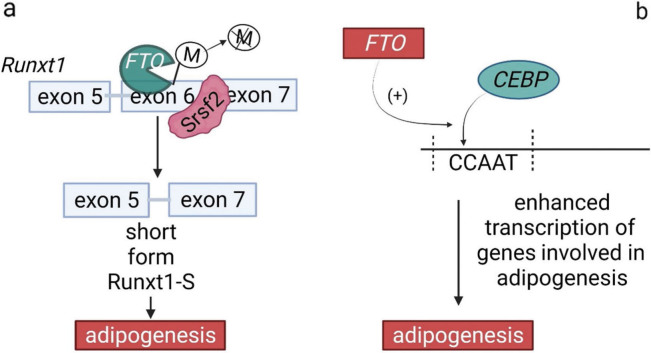
*FTO* regulation of genes involved in adipogenesis: a) schematic illustration of *FTO* demethylation activity on *Runxt1* gene and the impact on adipogenesis, and b) *FTO* binds to CEBP factors and enhances their binding activity to promote transcription of the genes responsible for adipogenesis. Runxt1=runt-related transcription factor 1, Srsf2=splicing regulatory proteins, CEBP*=*CAAT-enhancer-binding proteins (CAAT EBP)

Indirect regulation of lipid metabolism is evident in the interaction of the *FTO* gene with the runt-related transcription factor 1 (*Runxt1*) gene. Splicing regulatory proteins (Srsf) are responsible for the formation of a long isoform of the *Runxt1* gene. Srsf-binding factor overlaps with the substrate of the *FTO*. Under these conditions, *Runxt1-L* (long form) containing exons 5, 6 and 7 is not translated. When *FTO* removes a methyl group from exon 6, Srsf skips the exon 6 and the shorter isoform, Runxt1-S isoform, which is responsible for adipogenesis, is formed (Fig. 4a) (*54*).

Due to the possibility of binding to the CAAT (CAAT-enhancer-binding proteins – CAAT EBP) protein family, the *FTO* gene participates in the adipogenesis and modulates hypothalamic expression (*28*). CAAT proteins are transcription factors that can enhance binding activity to promoter regions and thereby regulate the expression of genes involved in adipogenesis (*CEBP* delta) or in fat cell differentiation (*CEBP* beta) (*55*). The *FTO* gene affects *CEBP* delta transcription by demethylating *N*^6^-methyldeoxyadenosine in the promoter of this gene (Fig. 4b) (*56*).

Another potential regulatory mechanism associated with the obesity and diabetes mellitus type 2 (DMT2) is a methylenetetrahydrofolate reductase (*MTHFR*) gene polymorphism. The *MTHFR* gene is located at 1p36.3, which correlates with neural tube defects, methylenetetrahydrofolate reductase deficiencies and vascular disease. The substitution of cytosine to thymine on nucleotide 677 causes the exchange of amino acid 222 from alanine to valine, also known as *C677T* mutation (*57*). Such substitution results in the inability to catalyse 5,10'-methyltetrahydrofolate to 5'-methyltetrahydrofolate due to high enzyme thermolability. The decreased enzyme activity is manifested by elevated homocysteine (Hcy) and reduced folate levels (*58*). Elevated Hcy levels have been associated with obesity as well as DMT2. A meta-analysis confirmed the correlation between *C677T* polymorphism and obesity. Moreover, obese participants had elevated Hcy levels with even higher levels when the risk allele was present (*59*). In addition, another study reported that the cumulative effect of *LEP* (leptin), *MTHFR* and *FTO* risk genotypes contributed to the highest BMI levels in humans (*60*).

### SNPs and connection to obesity

Although many studies have found that *FTO* gene polymorphisms are associated with *FTO* gene expression (*7*, *41*, *61*), there are many studies that reported that *FTO* gene polymorphisms are associated only with genes related to adipogenesis and not with *FTO* gene expression itself (*42*, *62*). This indicates that *FTO* has the ability to modulate genes besides itself through polymorphisms.

For example, Grunnet *et al*. (*42*) found that the *FTO* expression was not associated with the *FTO* rs9939609 genotype, neither in human skeletal nor in adipose tissues. Similarly, Smemo *et al.* (*62*) discovered that obesity-associated SNPs were not associated with *FTO* expression, but with the transcription factor Iroquois-class homeobox gene 3 (*IRX3*) and its expression in hypothalamic pro-opiomelanocortin neurons. *IRX3* gene expression level influences obesity by changing energy consumption and food intake (*63*, *64*).

The genotype *FTO* rs9939609 (T/A) has been found to be connected with increased expression of *FTO* and the hormone ghrelin which regulates digestive behaviour, and its increased expression leads to increased intake of dense food (food that has a higher number of calories per serving) (*41*). Sentinelli *et al*. (*61*) conducted a study with obese Italian individuals and found a strong positive relation between the *FTO* rs9939609 and rs9930506 SNPs and their BMI. Similarly, Scuteri *et al*. (*65*) found that the rs9930506 GG was associated with BMI and total body mass in more than 4000 subjects. The *FTO* rs9939609 risk variant was associated with brain malformations and structural atrophy. On the other hand, complete deficiency of *FTO* was lethal to humans (*47*). Higher BMI is associated with reductions in hippocampi (*66*) and global brain volume (*67*).

Polymorphisms associated with the *FTO* gene usually affect satiety responses, eating in absence of hunger, and loss of control (LOC) when overeating. LOC is accompanied by a daily increase in food intake and reduced feelings of satiety. This phenotype is observed in SNP risk allele carriers. For example, in the case of *FTO* rs9939609 polymorphism, postprandial satiety is reported to be 17.2% lower in risk allele carriers (*68*). Furthermore, the risk allele A was associated with eating in absence of hunger; however, there was no connection with living conditions (*69*). A summary of the characteristics of each FTO polymorphism indicating expected phenotypic traits in risk allele carriers is provided in Table 1 (*35*, *41*, *47*, *49*, *50*, *52*, *61*, *65*, *68*-*81*).

**Table 1:** Characteristics of most common *FTO* polymorphisms associated with obesity

*FTO* polymorphism				
Association	rs9939609 AA	rs9930506 GG	rs1421085 CC	rs8050136 AA
BMI	Higher (*61*)	Higher (*61*, *65*)	Higher (*49*, *50*)	Higher (*70*)
IRX	N/A	N/A	ARIDB5 repressor (*50*)	N/A
RPGRIP1L	N/A	N/A	Lower affinity for CUX1 (*49*, *50*)	Lower affinity for CUX1 (*49*, *50*)
FTO expression	Higher (*41*)	N/A	N/A	HCNE (*52*)
PP satiety	Lower (*71*)	N/A	N/A	N/A
Overeating	Yes (*68*, *69*)	N/A	N/A	N/A
Fat consumption	Higher (*72*–*75*)	N/A	N/A	Higher (*76*)
Carbohydrate consumption	N/A	Higher (*35*)	N/A	Lower (*76*)
Protein consumption	Higher (*77*)	Higher if AA/AG allele (*35*)	Higher (*35*)	N/A
Vitamin B_12_	N/A	N/A	N/A	Lower (*78*)
Lipids	Higher triglycerides, total cholesterol, HDL (*79*, *80*)	N/A	N/A	N/A
Birth mass	Higher (*81*)	N/A	N/A	N/A
Brain malformations	Yes (*47*)	N/A	N/A	N/A
Insulin, HOMA-IR	Higher (*80*)	N/A	N/A	Higher (*70*)
N/A=not available; BMI=body mass index, IRX=Iroquois homeobox gene, RPGRIP1L=retinitis pigmentosa GTPase regulator-interacting protein-1-like, PP satiety=postprandial satiety, HOMA-IR=homeostatic model assessment for insulin resistance, ARID5B=AT-rich interaction domain 5B, CUX1=cut-like homeobox 1, HCNE=highly conserved noncoding elements, HDL=high-density lipoprotein				

The influence of rs9939609 SNP on LOC is reported to be in 34.7% AA/AT subjects and in 18.2% TT subjects (*72*). These results suggest that the presence of risk alleles has an impact on assessing fullness, as in these studies, and consumption of more energy from fat. Another reason may be that the *FTO* polymorphisms affect neural responses when we consider physical expression. To support these statements, many studies have linked rs9939609 with an increase in fat intake. A 3-day study in children (*73*) reported higher total energy intake and higher fat intake without significant effects on carbohydrate and protein intake. Another study reported similar results, where they observed increased fat intake in risk allele carriers (*74*). A correlation between the increased fat intake and A allele was also observed (*75*). One study reported increased protein intake in individuals with rs9939609 variation (*77*). The results of these studies strengthen the evidence of the influence of *FTO* polymorphisms on energy homeostasis and feeding behaviour. In a study by den Hoed *et al.* (*71*) on postprandial satiety with the rs9939609 variation, results showed an important connection between the postprandial responses and lower satiety in individuals with the risk allele (Table 1). In rs9939609 risk allele carriers, there are differently methylated sites associated with different genes involved in the regulation of telomere length, nuclear factor kappa light chain enhancer of activated B cell (NF-κB) activity, and transcriptional regulation (*82*).

A similar effect on energy metabolism is observed in *FTO* rs8050136 risk allele A where it is correlated with higher energy expenditure from fat and lower from carbohydrates (*76*) and associated with higher total energy intake (*75*). One study reported higher protein intake, while others reported higher fat intake with weak or no effect on carbohydrate and fibre intake, suggesting that the main rs9939609 *FTO* polymorphism effect is higher fat and total energy intake with reduced postprandial satiety. Incorporating all these results, it is evident that polymorphisms play an important role in functionally effecting hunger and satiety response, although the mechanisms are not yet fully identified.

*FTO* expression in the hypothalamus may compromise food intake and the satiety response. Modulating energy homeostasis with recognition of essential amino acid deprivation and an additional direct impact on adipogenesis contribute to *FTO* overexpression. In support of this, there is a positive correlation between the *FTO* expression and increased BMI, but no association between the energy expenditure and physical activity (*24*), and increased levels of *FTO* mRNA and adiposity (*83*).

Obesity is highly associated with dyslipidaemia and increased risk of cardiovascular disease (CVD), with the possibility of an *FTO*-mediated effect on lipids. The research of Dorling *et al.* (*84*) revealed no effect of rs9939609 on lipids. In contrast, the results of Doney *et al*. (*79*) found an association between the risk allele rs9939609 and higher triacylglycerol (TG) levels. The main difference between these two studies is that in the study that found no association with lipids and polymorphisms, samples were collected shortly after eating and then measured. Study design limitation could lead to differences in the results, as lipid concentrations may have taken longer to change in a way that could contribute to vascular disease. Also, with respect to altered lipid concentrations, the time period over which lipids were altered should be considered. A large meta-analysis has confirmed the association with the rs9939609 A variant and CVD (*85*). Future studies should include more laboratory tests, larger samples and multiple genotyping with various epigenetic factors such as lifestyle to better understand this complex mechanism and possibly discover new variants.

Thus, most studies indicate that certain *FTO* gene polymorphisms affect appetite change and food intake, leading to mass gain and obesity. A higher consumption of certain food may be associated with a particular polymorphism. Individuals caring the *FTO* rs9930506 risk variant are expected to have higher levels of protein and carbohydrate intake with an upregulation of *FTO* and downregulation of *IRX3* expression (*86*). According to these findings, it is possible to conclude that a particular polymorphism can cause a change in dietary habits and expression that leads to obesity development. Indeed, lifestyle changes in the form of food intake and physical activity alter the previous influence of diet impact in risk allele carriers (*87*). A recent study has confirmed that individuals with extra mass and *FTO* rs9939609 risk genotype had higher levels of BMI, total cholesterol, insulin, high-density lipoprotein (HDL) and homeostatic model assessment for insulin resistance (HOMA-IR) (*80*). Another study reported an association with fatty acid intake and *FTO* expression in adipose tissue (*88*). In addition, a recent study has reported a 2.5-fold higher risk of overweight or obesity when having a high dietary inflammatory index (DII) in the carriers of rs9939609 risk allele, along with other complexes (*89*). All this suggests there are consequences of the diet type and nutrient-gene interactions. One approach to treating obesity using epigenetic engagement is a diet rich in vitamins, *e.g*. niacin, vitamin B_12_, curcumin and catechin along with anti-inflammatory minerals such as zinc and selenium. Curcumin and catechin, especially vitamin B_12_, are discussed in the next section.

### Epigenetic factors

Different exogenous and endogenous factors have a major impact on modifying gene expression. They may influence transcriptional activity through the commonly accepted mechanism of methylation or hypomethylation of CpG islands. Unexpected epigenetic factor is cow’s milk that provides substantial amount of microRNA, especially mircoRNA-29b (miRNA-29b) and microRNA-29s (miRNA-29s). The presented hypothesis is that miRNA-29b targets mRNA of branched-chain α-ketoacid dehydrogenase (BCKD) and downregulates branched-chain aminoacyl (BCAA) catabolism, which could explain increased levels of BCAA in serum. *FTO* is BCAA sensor and brings essential amino acids to mechanistic target of rapamycin complex 1 (mTORC1), leading to hiperactivated mTORC1 signalling and insulin resistance. This thesis puts milk as one of the overlooked regulators of potential epigenetic signalling mechanism that may represent a new point of obesity treatment. Also, it has been shown that miRNA-29b and microRNA-21 (miRNA-21) targeting can indirectly downregulate mRNA of the DNA methyltransferase (DNMT) affecting methylation rates and thereby leading to FTO overexpression, ultimately causing obesity (*90*-*92*).

**Fig. 5:**
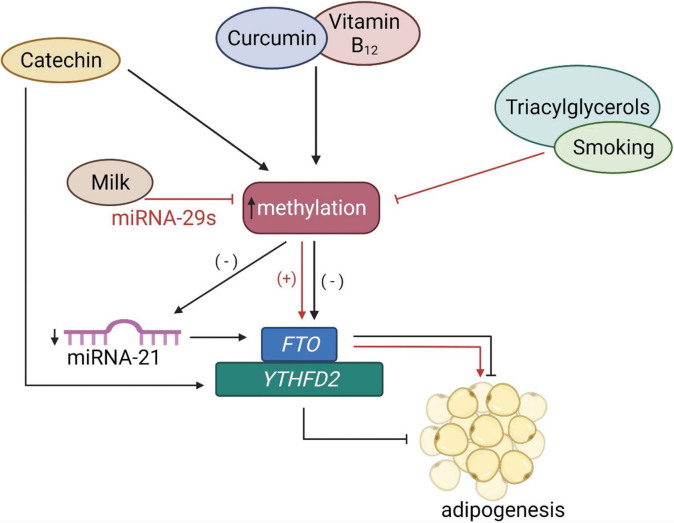
Epigenetic regulation *via* the *FTO* gene. External factors such as vitamin B_12_, curcumin or green tea increase methylation of the *FTO* gene and block adipogenesis. On the other hand, milk, triacylglycerols and smoking inhibit methylation and thereby increase the *FTO* expression, causing adipogenesis. Catechin also increases YTHFD2 activity, which is m^6^A reader, therefore YTHDF2 activity increases methylation levels of the *FTO* gene and blocks adipogenesis. *FTO*=fat mass and obesity-associated gene, YTHDF2=YTH *N*^6^-methyladenosine RNA-binding protein 2, miRNA-29s=microRNA-29 family members, microRNA-29b=member of the microRNA-21 family, miRNA-21=microRNA-21

Cow’s milk has negative effects in two ways simultaneously: (*i*) increased BCAA levels lead to *FTO* overexpression, and (*ii*) suppression of DNMT contributes to hypomethylation of CpG sites, and again leads to *FTO* overexpression. Both mechanisms ultimately lead to increased translation levels and activated mTORC1. Milk enhances mechanisms necessary for cell proliferation and adipogenesis. The use of fermented products such as yogurt, acidophilus and fermented cheese has the opposite effect. In addition, they contain a significant amount of vitamins B_2_ and B_12_. Epigenetic regulation *via* the *FTO* gene is shown in Fig. 5.

Vitamin B_12_ is a micronutrient of great importance for human metabolism and it has been reported that its supplementation influences methylation of genes associated with adiposity, type 2 diabetes, insulin resistance and other metabolic abnormalities. Vitamin B_12_ deficiency is associated with diminished methylation of homocysteine (Hcy). In the production of S-adenosyl methionine (SAM), a methyl donor, B_12_ is required for adequate methylation (*93*). It is also known to play an important role in foetal and neural development.

Recent studies report a potential role in gestational diabetes mellitus (GDM), where it may have an impact on foetal development. Low B_12_ concentrations are associated with obesity and insulin resistance in pregnant women, which increases the risk of GDM and influences foetal metabolic abnormalities later in life, such as higher risk for obesity and impaired insulin response (*94*). In addition, the *FTO* variants rs8050136 and rs2388405 are associated with lower B_12_ levels (*78*). Recent findings report that B_12_ supplementation affects methylation and primarily reduces the expression of miR21 (microRNA 21) and secondarily of the *FTO* and other genes involved in DMT2 pathways (*94*). These results are important for healthy mass management, which may have global implications.

Dietary supplementation with curcumin has been reported to reduce aspartate aminotransferase (AST), lactate dehydrogenase (LDH), cholesterol, triacylglycerols, *FTO* and YTH *N*^6^-methyladenosine RNA-binding protein 2 (YTHDF2) mRNA expression and increase m^6^A levels (*95*). YTHDF2, like *FTO*, recognizes m^6^A and can mediate RNA degradation and cell differentiation, thereby regulating mRNA stability.

In green tea, the action of catechin is manifested mainly in the reduction of *FTO* expression to increase methylation. It also acts on the YTHDF2 protein, which activates the decomposition of methylated mRNA and blocks adipogenesis (Fig. 5) (*96*). Gallocatechin is the most promising natural *FTO* inhibitor, as experiments showed a similarity of binding site along with a stronger affinity to orlistat, anti-obesity medicine, of more than 60% (*97*).

Lifestyle is one of the most important epigenetic contributors to the occurrence of obesity, where one or more genotypes can have the same or different expression localization. Various molecular interactions with other genes are another matter that requires further studies.

Gestational diabetes mellitus (GDM) is another complication of obesity. GDM is described as insulin resistance, usually diagnosed in the second or third trimester, resulting from insulin attenuation and diminished glucose metabolism. Risk factors for GDM include increased BMI, family history, older age, increased lipid levels, with diabetogenic hormones such as progesterone and prolactin contributing to the development of insulin resistance in pregnancy (*98*).

There are numerous external factors that influence expression through methylation. In one study, an inverse correlation was shown between the placental methylation levels of CpG 11 site and CpG 6, 7, 8, 9 on CpG island 1 of the *FTO* promoter and birth mass (*99*). Franzago *et al.* (*81*) reported a connection between rs9939609 and neonatal birth mass, and a lack of association between the *FTO* promoter methylation levels and GDM. Furthermore, CpG 1 site methylation levels are associated with smoking in GDM during pregnancy. Higher levels of triglycerides are associated with methylation of CpG 2. The positive correlation between the placental *FTO* mRNA expression and birth mass suggests an important regulation metabolism in the placenta. Hypomethylation of the *FTO* promoter along with the existing metabolic pathology (such as diabetes) contribute to altering foetal programming. Decreased methylation rates of CpG sites are additionally reported with a higher risk of developing diabetes in patients with DMT2 when methylation levels of the *FTO* are lower (*100*).

Food type selection may be associated with the *FTO* SNPs as individuals often choose food rich in fat and high-carbohydrate diets. In these terms, the *FTO* polymorphism represents an important genetic factor with a global impact on human health. The effects of epigenetic exposure should also be considered because the *FTO* encodes demethylase and it is subject to various external factors.

The finding that epigenetic mechanisms influence gene expression opens new perspectives on possibilities to treat obesity. This includes counselling on the lifestyle including diet, appropriate supplements and medications, and avoiding known substances that may have a negative effect on gene expression. And the beauty of the future is in revealing the multiple interactions of genes and the influence on the epigenome through the prism of exposome.

## CONCLUSIONS

The effect of *FTO* demethylation continues to be investigated. Positive effects of certain substances on the expression of the *FTO* gene, such as the intake of curcumin, green tea and vitamin B_12_ with lifestyle changes, as well as negative effects of environmental factors such as smoking and consuming food such as cow’s milk are known. Many diseases are associated with risk alleles of the *FTO* gene such as metabolic syndrome, diabetes, obesity and cancer. The solution is a new approach through epigenetic changes that can lead to reduced gene expression and a lower phenotypic predisposition to disease development. The interaction of individual genes with the *FTO* gene/protein and whether there is a pathway of action of the *FTO* gene should be considered, but also how *FTO* expression would affect other genes. Further research that will investigate the complete influence of the *FTO* gene is needed to better understand the underlying mechanisms associated with *FTO* gene polymorphisms, epigenetic regulation and food intake in humans.
